# Heat-Treatments Affect Protease Activities and Peptide Profiles of Ruminants' Milk

**DOI:** 10.3389/fnut.2021.626475

**Published:** 2021-03-10

**Authors:** Juliana A. S. Leite, Carlos A. Montoya, Simon M. Loveday, Evelyne Maes, Jane A. Mullaney, Warren C. McNabb, Nicole C. Roy

**Affiliations:** ^1^Riddet Institute, Massey University, Palmerston North, New Zealand; ^2^Smart Foods Innovation Centre of Excellence, AgResearch Limited, Palmerston North, New Zealand; ^3^Beyond Foods Innovation Centre of Excellence, AgResearch Limited, Lincoln, New Zealand; ^4^High-Value Nutrition National Science Challenge, Auckland, New Zealand; ^5^Liggins Institute, The University of Auckland, Auckland, New Zealand; ^6^Department of Nutrition, University of Otago, Dunedin, New Zealand

**Keywords:** plasmin, plasminogen, cathepsin D, bovine milk, ovine milk, caprine milk, peptides

## Abstract

Proteases present in milk are heat-sensitive, and their activities increase or decrease depending on the intensity of the thermal treatment applied. The thermal effects on the protease activity are well-known for bovine milk but poorly understood for ovine and caprine milk. This study aimed to determine the non-specific and specific protease activities in casein and whey fractions isolated from raw bovine, ovine, and caprine milk collected in early lactation, and to determine the effects of low-temperature, long-time (63°C for 30 min) and high-temperature, short-time (85°C for 5 min) treatments on protease activities within each milk fraction. The non-specific protease activities in raw and heat-treated milk samples were determined using the substrate azocasein. Plasmin (the main protease in milk) and plasminogen-derived activities were determined using the chromogenic substrate S-2251 (D-Val-Leu-Lys-pNA dihydrochloride). Peptides were characterized using high-resolution liquid chromatography coupled with tandem mass spectrometry. The activity of all native proteases, shown as non-specific proteases, was similar between raw bovine and caprine milk samples, but lower (*P* < 0.05) than raw ovine milk in the whey fraction. There was no difference (*P* > 0.05) between the non-specific protease activity of the casein fraction of raw bovine and caprine milk samples; both had higher activity than ovine milk. After 63°C/30 min, the non-specific protease activity decreased (44%; *P* > 0.05) for the bovine casein fraction only. In contrast, the protease activity of the milk heated at 85°C/5 min changed depending on the species and fraction. For instance, the activity decreased by 49% for ovine whey fraction, but it increased by 68% for ovine casein fraction. Plasmin and plasminogen were in general inactivated (*P* > 0.05) when all milk fractions were heated at 85°C/5 min. Most of the peptides present in heat-treated milk were derived from β-casein and α_S1_-casein, and they matched the hydrolysis profile of cathepsin D and plasmin. Identified peptides in ruminant milk samples had purported immunomodulatory and inhibitory functions. These findings indicate that the non-specific protease activity in whey and casein fractions differed between ruminant milk species, and specific thermal treatments could be used to retain better protease activity for all ruminant milk species.

## Introduction

Proteases are degradative enzymes found in plants, microorganisms, and animals; they hydrolyze proteins into peptides and amino acids (1). Proteases present in milk (plasmin, elastase, cathepsin D, and carboxy- and aminopeptidases) produce and control the hydrolysis of peptides before and during gastrointestinal digestion, promoting bioactivities (e.g., sleep induction, mucosal development, and immunomodulatory and gastrointestinal functions) and helping with digestion within the gastrointestinal tract (2–4). The latter is important for infant when the gastrointestinal tract is immature, and milk enzymes appear to help the infant to digest milk proteins.

Most of the proteases in bovine milk are secreted in their inactive form (zymogens) and become active by cleavage of a specific peptide bond by an activator (5). A balance between protease activators and inhibitors is essential to retain adequate peptide and amino acid profiles for optimal digestion, absorption, and function in the gastrointestinal tract (4).

Thermal processes used in the food industry can affect protease activities in milk (6). Most of the components (zymogens, active enzymes, activators, and inhibitors) in the proteolytic system are heat-sensitive and can be inactivated during ultra-high-temperature (UHT) conditions (e.g., 143°C for 15 s). However, mild treatments, such as pasteurization (e.g., 72°C for 15 s), do not completely inactivate some zymogens and their activators. Thus, mild treatments can result in higher protease activity in pasteurized milk when compared with their raw or UHT counterparts, due to destabilization of proteolytic systems and conversion of zymogens into an active form without the interference of inhibitors (6–9).

The protease activity and the thermal effects on the protease activity are well-known for bovine milk. For instance, bovine casein fraction has a higher concentration of plasmin and plasminogen than the bovine whey fraction (10). However, the protease activity and the thermal effect on the protease activity in other ruminant milk (ovine and caprine) fractions are little studied. Other ruminant milk species have different protein content, amino acid sequences, casein-to-whey protein ratio, and micelle structure to bovine milk (11). Given these differences, the study hypothesized that thermal treatments applied to milk from different ruminant species might yield different protease activities and peptide sequences.

Thus, this study aimed to determine protease activities in casein and whey fractions from bovine, ovine, and caprine raw milk samples collected in early lactation, and to investigate the effect of thermal treatments (63°C for 30 min and 85°C for 5 min) on protease activities within each fraction. The non-specific protease activities of bovine, ovine, and caprine skim milk samples, either raw or heat-treated, were determined using the substrate azocasein. Plasmin and plasminogen activities were determined using the chromogenic substrate S-2251 (D-Val-Leu-Lys-pNA dihydrochloride). Peptides were characterized by peptidomics using high-resolution liquid chromatography coupled with tandem mass spectrometry (LC-MS/MS).

## Materials and Methods

### Milk Sampling

Fresh raw bovine, ovine, and caprine milk samples were collected at early lactation to avoid the variation of enzyme activities due to lactation stage as reported previously (12). Early lactation was defined as the period between 14 and 50, 35, and 40 post-partum days for bovine, ovine, and caprine, respectively (12–14). Raw bovine milk was provided by Dairy 4 Farm, Massey University (Palmerston North, New Zealand), and ovine and caprine milk samples obtained from local dairy farms within 10 km to Palmerston North, New Zealand. Milk heat-treatments were carried out in the morning on the same day as collection. For each species, three batches of milk from different animals were collected and heat-treated over three separate days (i.e., *n* = 3 biological replicates).

### Heat-Treatments

Ruminant milk samples were first skimmed at 2455 *g* for 30 min at 4°C to remove the fat. Skim milk samples were then heat-treated at 63°C for 30 min and 85°C for 5 min using a thermostatically controlled water bath with shaking. These thermal conditions were chosen to represent the conventional pasteurization using low-temperature, long-time (LTLT; 63°C for 30 min) and high-temperature, long-time (HTLT; 85°C for 5 min) treatments. The heating was performed in a 150-ml capped sterile bottle, and the temperature was monitored through a thermometer (MS6514, Mastech, Dongguan, China) inserted at the center of the liquid. After holding the milk for the required temperature and time, the milk samples were rapidly cooled down in an ice-water bath until the temperature reached approximately 10°C. Raw and heat-treated samples were aliquoted and frozen at −20°C and analyzed within 1 week. Samples were frozen to avoid protein hydrolysis during cold storage (5°C) as previously reported (15, 16). Raw milk samples were used as controls.

### Preparation of Casein and Whey Milk Fractions

The casein and whey fractions were separated by ultracentrifugation to determine the protease activity of each fraction. Raw and heat-treated milk samples were thawed and ultracentrifuged at 100,000 × *g* for 1 h at 20°C. The supernatant (milk whey fraction) and pellet (casein micelles) were separately collected. The casein fraction was then prepared by suspending the casein micelles in pH 8.0 buffer (50 mM Tris-HCl, 110 mM NaCl, and 50 mM ε-amino-n-caproic acid) as previously described (5). The suspended casein fraction was incubated at room temperature for 2 h to dissociate plasmin and plasminogen from the casein micelles. The casein suspension was then ultracentrifuged at 100,000 × *g* for 1 h at 20°C, and the supernatant (or casein fraction) was used to determine the non-specific protease, plasmin, and plasminogen-derived activities in raw and pasteurized (63°C/30 min and 85°C/5 min) ruminant's milk.

### Determination of Non-specific Protease Activity

Non-specific protease activity of both casein and whey fractions isolated from raw and heated ruminant milk samples were determined as previously described (17–19) using azocasein (a protein coupled with diazotized aryl amines) as substrate.

Briefly, 1 ml of 1% azocasein solution (catalog A2765, Sigma-Aldrich, St. Louis, USA) was combined with 100 μl of casein or whey fraction. Samples were incubated at 36°C for 15 min, and the reaction was stopped by the addition of 2 ml of 5% TCA solution (catalog T6399, Sigma-Aldrich, St. Louis, USA). Samples were centrifuged at 2455 *g* at room temperature for 4 min, and the supernatant was filtered on paper Whatman 1 (Sigma-Aldrich, St. Louis, USA). The absorbance of the filtrate was read at 345 nm in a UV/Visible spectrophotometer (Genesys, Thermo Fisher Scientific, Massachusetts, USA).

Absorbance values were compared to a standard calibration curve, which was generated by mixing 100 μl of protease from *Bacillus* sp. solution (16 U/g, catalog P3111, Sigma-Aldrich, St. Louis, USA) at different concentrations with 1% azocasein solution. The absorbance was determined as described above at 345 nm, and the coefficient of determination (*r*^2^) obtained was 0.9991. The analysis was performed in triplicate.

### Determination of Plasmin and Plasminogen Activities

Plasmin (and its zymogen plasminogen) is the major enzyme found in bovine milk. In this study, plasmin was chosen as a model milk enzyme to compare its activity across ruminant milk samples and thermal processes. The plasmin and plasminogen-derived activities naturally present in casein and whey milk fractions were determined in all samples using the method previously described (20, 21).

Plasminogen was converted into active plasmin during a 60-min incubation at 37°C of 500 μl of milk fraction (casein or whey) in the presence of 500 μl of urokinase solution (catalog U4010, Sigma-Aldrich, St. Louis, USA; 200 Ploug U/ml prepared with 100 mM Tris–HCl, pH 8).

Plasmin and plasminogen-derived activities, after plasminogen activation by urokinase, were determined by measuring the rate of hydrolysis of chromogenic substrate S-2251 (H-D-valyl-L-leucyl-L-lysine-*p*-nitroanilide dihydrochloride, catalog V0882, Sigma-Aldrich, St. Louis, USA) and release of *p*-nitroanilide. The method involves the reaction of 100 μl of the sample with 100 μl of buffer (100 mM Tris-HCl, 4 mM S-2251).

The absorbance at 405 nm of the milk samples was measured at 30-min intervals for 120–180 min, depending on the level of plasmin activity in the sample, in a multi-mode microplate reader (FLUOstar Omega, BMG Labtech, Ortenberg, Germany) at 37°C. The plasminogen-derived activity was calculated as the difference between the plasmin activity before and after plasminogen activation with urokinase. Plasmin activity was expressed in *p*-nitroanilide units (micromoles of *p*-nitroanilide released per minute) per milliliter of milk. The analysis was performed in triplicate.

### Peptide Characterization via LC-MS/MS

#### Peptide Extraction

The raw and heat-treated ruminant milk samples were analyzed by LC-MS/MS to determine the peptide sequences as described elsewhere (22, 23). Milk samples (500 μl) were subjected to acid precipitation with HCl (pH 4.5) at room temperature for 1 h. The milk samples were centrifuged at 14,000 × *g* for 25 min at 4°C. An aliquot of whey was taken from the middle of the supernatant. After removing the remaining supernatant, casein (pellet) was obtained. This fractionation method was different from the method used above, as here it was not required to dissociate the enzymes from casein micelles. This fractionation was conducted to be able to identify peptides with low abundance.

Casein was dissolved in 100 μl of 5% acetonitrile using ultrasonication. Following centrifugation, at 14,000 × *g* for 25 min at 4°C, 100 μl of whey and casein fractions was ultrafiltered individually using 10-kDa NanoSep centrifugal ultrafilters (Pall, Ann Arbor, Michigan, USA). The ultrafiltrates were then dried in a vacuum centrifuge and resuspended in 100 μl of 0.1% formic acid.

#### Data Acquisition

Mass spectrometry was carried out on a nanoflow Ultimate 3,000 UPLC (Thermo Scientific, San Jose, CA) coupled to Impact II mass spectrometer with a CaptiveSpray source equipped with a nanoBooster device (Bruker Daltonik, Bremen, Germany) operated at 1,800 V. For each fractionated sample, 1 μl of the sample was loaded on a C18 PepMap100 nano-Trap column (300 μm ID × 5 mm, 5 micron 100 Å) at a flow rate of 3,000 nl/min. The trap column was then switched in line with the analytical column ProntoSIL C18AQ (100 μm ID × 150 mm 3 micron 200 Å) (nanoLCMS Solutions, Gold River, CA). The reverse-phase elution gradient was from 2 to 20% to 45% solvent B over 60 min, total 88 min at a flow rate of 600 nl/min. Solvent A was LCMS-grade water with 0.1% formic acid; solvent B was LCMS-grade acetonitrile with 0.1% formic acid.

The fractionated samples were measured in data-dependent MS/MS mode, where the acquisition speed was 2 Hz in MS and 2–32 Hz in MS/MS mode depending on precursor intensity. Ten precursors were selected in the m/z 150–2,200 range, with one to eight charged peptides selected. The analysis was performed in positive ionization mode with a dynamic exclusion of 60 s.

#### Peptide Identification

The PEAKS × Studio data analysis software package (Bioinformatics Solutions Inc, Waterloo, Canada) was used to analyze the LC-MS/MS data. The raw data were refined by a built-in algorithm that allows the association of chimeric spectra. The peptides were identified with the following parameters: a precursor mass error tolerance of 10 ppm and fragment mass error tolerance of 0.05 Da were allowed; peptides with a length starting at four amino acids long were included; the UniProt *Ovis aries* database (v2019.08, 27,855 sequences), UniProt *Bos taurus* database (v2019.08, 46,707 sequences), and UniProt *Capra hircus* database (v2019.08, 35,307 sequences) were used; and no enzyme was specified as a digestive enzyme. Oxidation (M), phosphorylation (STY), and deamidation (NQ) were chosen as variable modifications in Peaks DB, and unexpected modifications were accounted for in the Peaks PTM search module. A maximum of three post-translational modifications (PTMs) per peptide was permitted. False discovery rate (FDR) estimation was made based on decoy fusion. An FDR of <1% with a peptide spectrum match hit and a PTM A-score of 100 was considered adequate for confident peptide identification. The peptides identified in each fraction were then compiled for each milk type and heat-treatment combination. When peptides with the same amino acid sequences and retention time (i.e., redundant peptides) were identified multiple times in the same sample, it was considered as only one peptide. The mass spectrometry data have been deposited to the ProteomeXchange Consortium via the PRIDE partner repository with the dataset identifier PXD022702 (http://proteomecentral.proteomexchange.org/cgi/GetDataset?ID=PXD022702) (24).

#### Comparison of Peptide Profiles Between Treatments for Each Species

The identified non-redundant peptides were used to identify common peptides between raw and 63°C/30 min, and raw and 85°C/5 min treatments within the species to identify resistant peptides to each thermal treatment.

#### Enzyme Prediction

The EnzymePredictor tool (http://bioware.ucd.ie/~enzpred/Enzpred.php) (25) was used to predict which proteases were involved in peptide cleavage for each milk type and heat-treatment combination. The tool was built to identify the action of 35 enzymes, which are from endogenous, bacterial, and digestive system sources. Here, only the activity of main native enzymes in milk (plasmin, cathepsin D, and elastase) were identified. The non-redundant peptide sequences organized by treatment and species combinations were uploaded on the EnzymePredictor tool. For each enzyme, the activity was predicted based on the number of peptides cleaved (Figure 4).

#### Bioactive Peptides Prediction

The non-redundant peptide sequences in each treatment and species were used to predict the potential bioactive properties using the Milk Bioactive Peptide Database (MBPDB) (http://mbpdb.nws.oregonstate.edu/) (26). The MBPDB compiles a comprehensive database of functional peptides in milk from mammalian species across the available literature sources, allowing comparison of known functional peptides with biological datasets to explore the presence of potential bioactive peptides in food sources.

In this study, the query batch of peptide sequences for each treatment and species was set to find the exact match in the MBPDB database without distinction of species. A list of bioactive peptides was obtained for each sample to identify potential bioactive peptides and compare them between treatments within the same species.

### Statistical Analysis

Statistical analyses were performed using the Mixed Model procedure of SAS (SAS/STAT version 9.4; SAS Institute Inc., Cary, NC, USA). A 3 × 2 × 2 factorial model was used to test the effect of milk treatments (raw, 63°C/30 min and 85°C/5 min), fractions (casein and whey), species (bovine, ovine and caprine) on non-specific protease, plasmin, and plasminogen activities.

The model diagnostics for each response variable were tested after combining the Output Delivery System Graphics procedure and the Repeated statement of SAS, before comparing the means. The repeated statement in the Mixed Model procedure was used to test the homogeneity of variances by fitting models with the Restricted Maximum Likelihood method and comparing them using the log-likelihood ratio test. Each response variable in the selected model had adjusted equal variances across treatments. Selected means were compared using adjusted Tukey test when the *F* value of the analysis of variance was significant (*P* < 0.05).

## Results

### Heat-Treatments Affect Non-specific Protease Activity in Ruminant Milk Fractions

**Figure 1:**
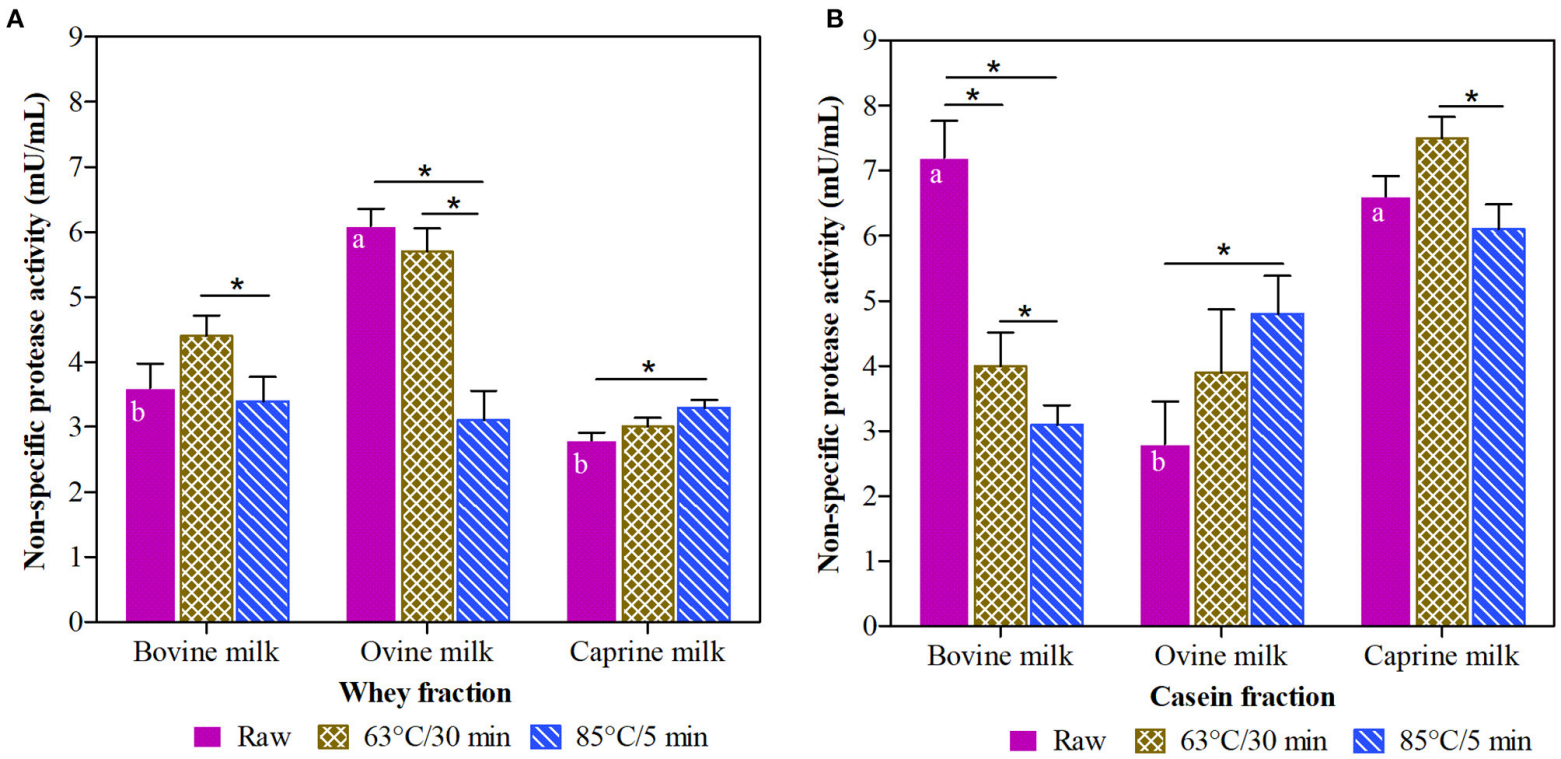
Non-specific protease activity in whey **(A)** and casein **(B)** fraction of ruminant milk samples (bovine, ovine, and caprine) unheated (raw) and heated (63°C/30 min and 85°C/5 min). There was a significant effect (*P* < 0.001) for all main factors (species, treatments, and fractions) and all their interactions. Values are means ± SEs, *n* = 3. Means with different letters across species (bovine, ovine, and caprine) within raw milk for each fraction differ significantly (*P* < 0.001). Lines with an asterisk represent significant differences (*P* < 0.05) between treatments (raw, 63°C/30 min and 85°C/5 min) within each species. For raw bovine milk and raw and heated (63°C/30 min and 85°C/5 min) ovine and caprine milk, there were differences between the whey and casein fraction (*P* < 0.001).

This study reports the effect of different thermal treatments (raw, 63°C/30 min and 85°C/5 min) on non-specific protease activity of casein and whey fractions isolated from ruminant milk (bovine, ovine, and caprine) samples (Figure 1). There was a significant interaction between species, heat-treatments, and fractions (*P* < 0.001).

Raw milk from bovine and caprine species had higher non-specific protease activity in the casein than whey fraction. For example, raw caprine milk showed 48% higher non-specific protease activity in casein than whey fraction. However, in the whey fraction, the non-specific protease activity of raw ovine milk samples showed, on average, 25% higher (*P* < 0.05) activity than raw bovine milk and raw caprine milk samples (Figure 1A).

The intensity of the thermal treatments applied to ruminant milk samples affected the non-specific protease activity differently in whey and casein fractions. Compared with their raw fractions, the non-specific protease activity in the bovine casein fraction was reduced (*P* < 0.05) by 44 and 71% after the thermal treatments at 63°C/30 min and 85°C/5 min, respectively (Figure 1B), whereas, these thermal treatments did not affect the non-specific protease activity of the bovine whey fraction (Figure 1A).

For the whey fraction of ovine milk, the thermal treatment at 63°C/30 min was more effective in retaining the non-specific protease activity compared to 85°C/5 min. For raw caprine milk, neither of the thermal treatments significantly affected (*P* > 0.05) the non-specific protease activity in the casein fraction (Figure 1B). From the raw fractions, the thermal treatment at 85°C/5 min significantly increased (*P* < 0.05) the non-specific protease activities in casein fraction of ovine (68%) (Figure 1B) and whey fraction of caprine (12%) milk (Figure 1A).

### Heat-Treatments Affect Plasmin and Plasminogen-Derived Activity in Ruminant Milk Fractions

**Figure 2:**
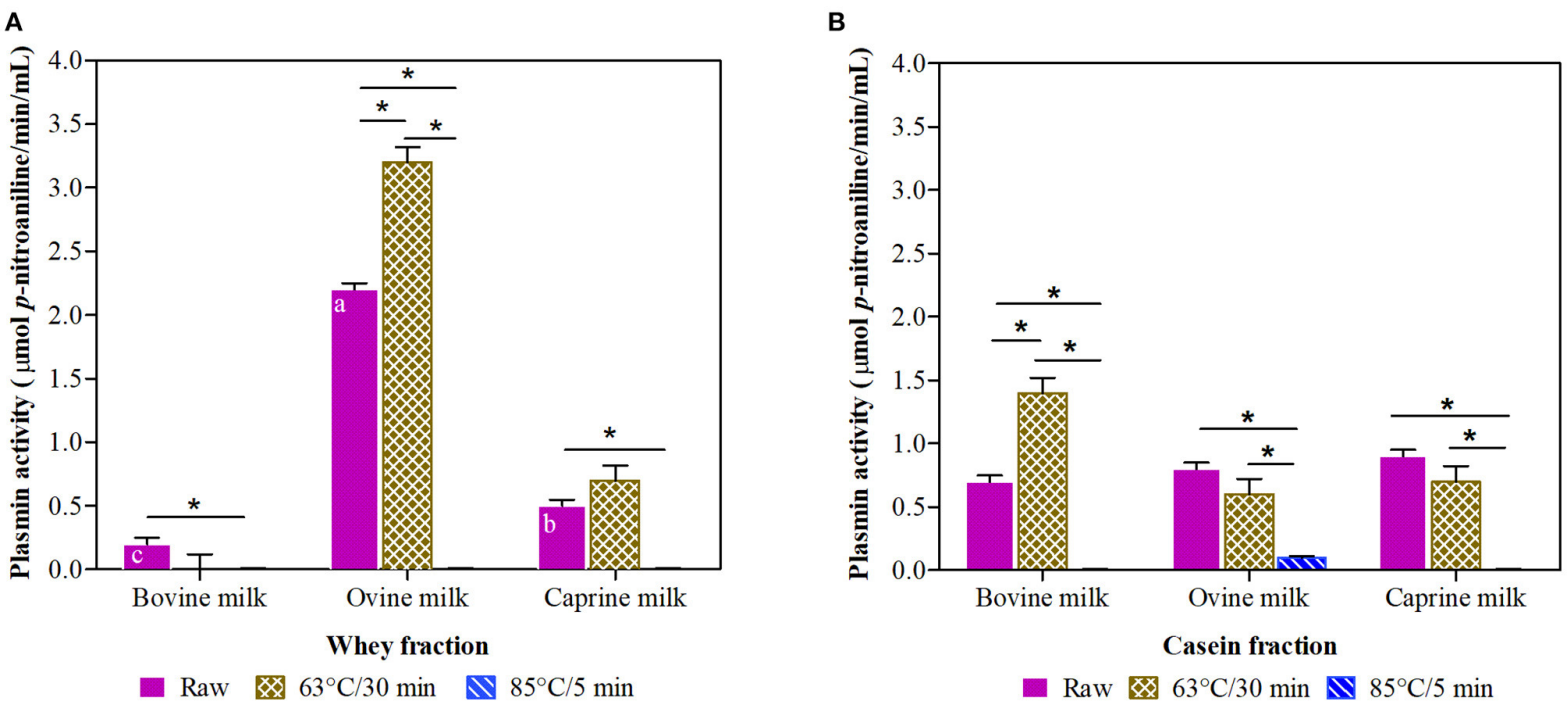
Plasmin activity in whey **(A)** and casein **(B)** fraction of ruminant milk (bovine, ovine, and caprine) unheated (raw) and heated (63°C/30 min and 85°C/5 min). There was a significant effect (*P* < 0.001) for all main factors (species, treatments, and fractions) and all their interactions. Values are means ± SEs, *n* = 3. Means with different letters across species (bovine, ovine, and caprine) within raw milk for each fraction differ significantly (*P* < 0.001). Lines with an asterisk represent significant differences (*P* < 0.05) between treatments (raw, 63°C/30 min and 85°C/5 min) within each species. For raw caprine and raw and heated at 63°C/30 min bovine and ovine milk, there were differences between the whey and casein fraction (*P* < 0.001).

Raw ovine whey fraction had 4-fold and 11-fold higher plasmin activity (Figure 2A) than their matched raw caprine and bovine whey fractions, respectively.

The thermal treatment at 85°C/5 min of the raw milk resulted in a significant decline (93 to 100%) of plasmin activity in both whey and casein milk fractions of all species (Figures 2A,B). In contrast, the thermal treatment at 63°C/30 min resulted in significantly increased plasmin activity in ovine whey and bovine casein fractions (45 and 100% increase, respectively).

**Figure 3:**
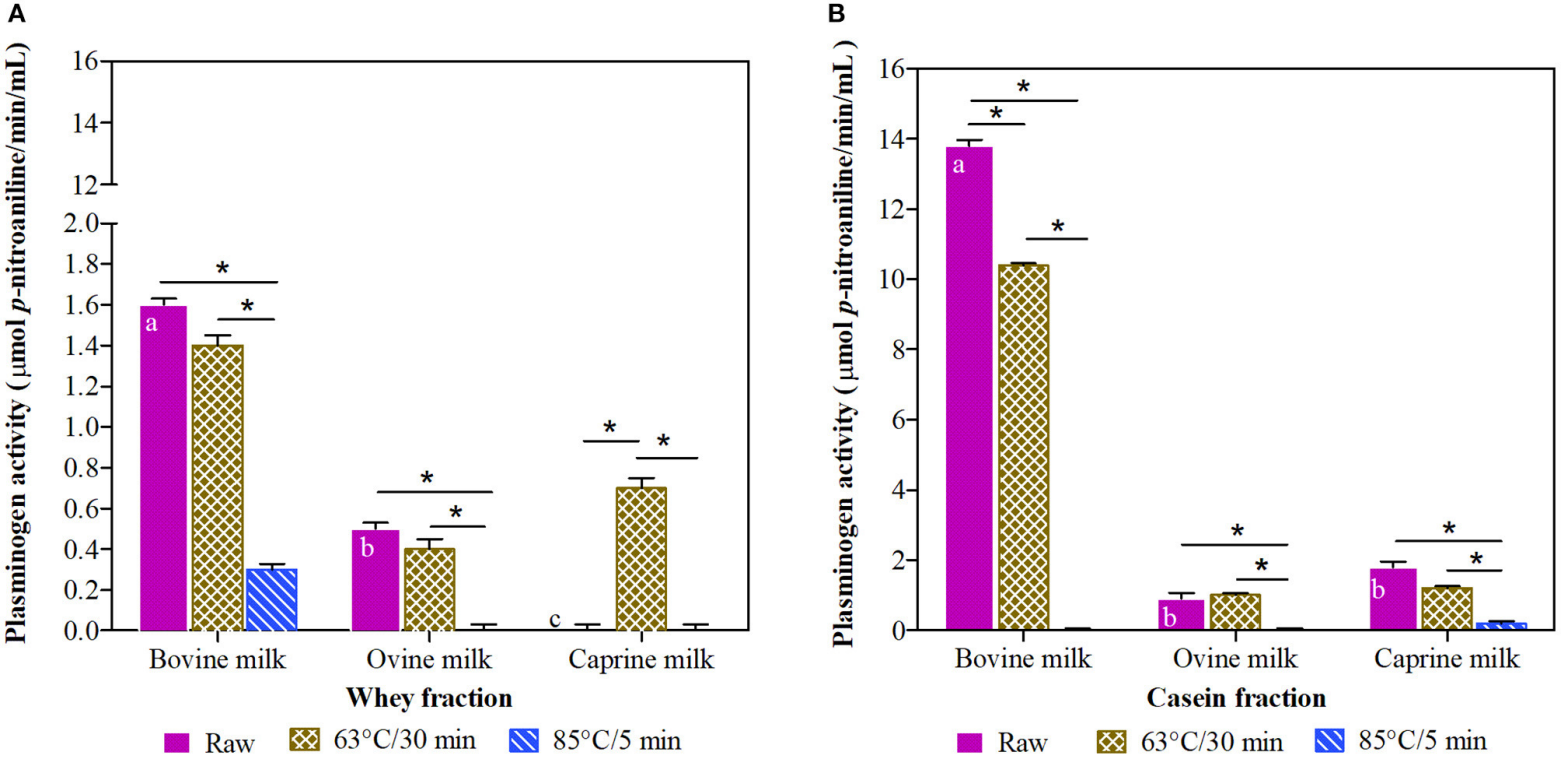
Plasminogen activity in whey **(A)** and casein **(B)** fraction of ruminant milk (bovine, ovine, and caprine) unheated (raw) and heated (63°C/30 min and 85°C/5 min). There was a significant effect (*P* < 0.001) for all main factors (species, treatments, and fractions) and all their interactions. Values are means ± SEs, *n* = 3. Means with different letters across species (bovine, ovine, and caprine) within raw milk for each fraction differ significantly (*P* < 0.001). Lines with an asterisk represent significant differences (*P* < 0.05) between treatments (raw, 63°C/30 min and 85°C/5 min) within each species. For raw and heated (63°C/30 min and 85°C/5 min) bovine milk, raw and heated at 63°C/30 min caprine milk, and heated at 63°C/30 min ovine milk, there were differences between the whey and casein fraction (*P* < 0.001).

Among raw milk samples, the bovine milk had the highest (*P* < 0.05) plasminogen-derived activity in whey and casein fractions, with a 9-fold higher concentration in the casein fraction (Figures 3A,B). In general, the milk fractions heated at 63°C/30 min had a similar plasminogen-derived activity compared to their raw counterparts, with the exception of caprine whey and bovine casein fractions. In contrast and in general, the treatment at 85°C/5 min reduced the plasminogen-derived activity up to 100%.

### Differences in Endogenous Peptide Profiles and Their Predicted Bioactivity Between Raw and Heat-Treated Ruminant Milk Samples

The peptide sequences that were identified in both raw and 63°C/30 min, and raw and 85°C/5 min, milk samples for each species are shown in Supplementary Tables 1, 2. For raw bovine milk, 72 non-redundant peptide sequences were identified, but only three and five of them were also detected after 63°C/30 min and 85°C/5 min, respectively. Other peptides were observed for 63°C/30 min (129) and 85°C/5 min (125).

For ovine milk, there were 95 and 41 common peptide sequences between raw and milk heated at 63°C/30 min and 85°C/5 min, respectively. However, in contrast with bovine milk, the number of non-common peptide sequences between the treatments decreased 13–61% at 63°C/30 min and 39–83% at 85°C/5 min in caprine and ovine milk samples, respectively.

The non-redundant peptide sequences identified in each treatment within the species were analyzed with the EnzymePredictor to predict which milk proteases (plasmin, cathepsin D or elastase) were involved in peptide bond cleavage during the thermal treatments. Similarly, the Milk Bioactive Peptide Database (MBPDB) was used to predict whether the identified peptide sequences in each milk sample matched any peptides reported with bioactivity.

**Figure 4:**
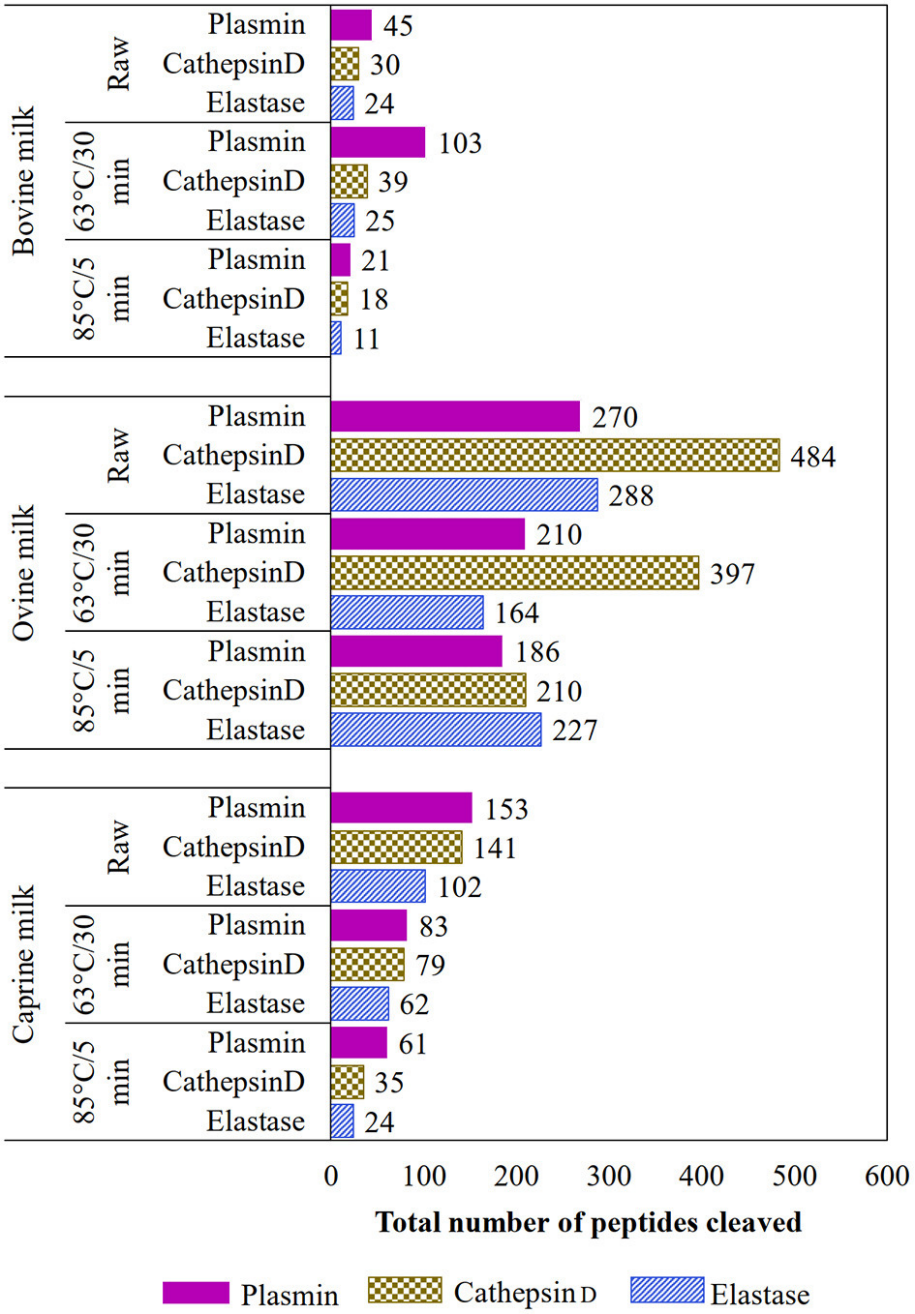
Total predicted number of non-redundant peptides attributed to plasmin, cathepsin D, or elastase in raw and heated (63°C/30 min and 85°C/5 min) ruminants' milk (bovine, ovine, and caprine) using the EnzymePredictor tool.

Based on the EnzymePredictor, plasmin was the main protease involved in the protein hydrolysis of heat-treated bovine and caprine milk samples (Figure 4). However, for ovine milk, the enzyme cathepsin D (and elastase for 85°C/5 min) was most likely involved in the protein hydrolysis. In general, the intensity of the heat-treatment affected the hydrolysis of proteins, and the use of high-temperature, short-time (85°C/5 min) treatment showed a lower number of peptides cleaved by the three analyzed endogenous enzymes (plasmin, cathepsin D, and elastase) compared with raw and lower-temperature, long-time (63°C/30 min) treatment, at least for bovine and caprine milk types.

The MBPDB revealed that the heat-treatments affected the presence of potential bioactive peptides in ruminant milk samples. For example, raw bovine milk showed a low number (3) of potential bioactive peptides compared with heat-treated milk samples at 63°C/30 min (5) (Supplementary Table 3). The potential bioactive peptides identified in bovine milk samples have several purported functions including immunomodulatory, antimicrobial, inhibitory, and cytotoxic. In contrast, for ovine and caprine milk samples, there was a reduction (42–67%) of potential bioactive peptides when the raw milk samples were heat-treated at 63°C/30 min (Supplementary Table 3).

## Discussion

Studies investigating the distribution of protease activities between milk fractions have been mainly focused on bovine milk, and there is little information reported for caprine and ovine milk. This study is the first to show the effect of different thermal treatments on endogenous protease activities of milk fractions from different ruminant species. In this study, milk casein and whey fractions isolated from bovine, ovine, and caprine milk samples were used to determine differences related to species, and the effect of different thermal treatments (63°C/30 min and 85°C/5 min) on non-specific and specific protease (plasmin) activities. Peptide sequences in raw and heat-treated ruminant milk samples were also determined by high-resolution LC-MS/MS to predict both the native enzymes (plasmin, elastase, and cathepsin D) involved in peptide formation (protein hydrolysis) and potential bioactivity of the identified peptides.

The activity of all native proteases, shown as non-specific proteases, was similar between raw bovine and caprine milk samples, but lower than raw ovine milk samples in the whey fraction. The higher non-specific protease activity observed in raw ovine whey fraction could be associated with the action of cathepsin D, as predicted by the EnzymePredictor, which is the second proteinase with higher activity in milk, and it is normally present in acid whey (27).

The high plasmin activity in raw whey ovine milk samples compared to that in whey bovine and caprine milk samples could be explained by its lower casein:whey ratio (4.7, 3.1, and 3.5 in bovine, ovine, and caprine) and higher protein content (1.6-fold and 1.4-fold higher than bovine and caprine milk) (28).

Thermal treatments are commonly applied to inactivate potential microbial pathogens in manufacturing dairy products and extend their shelf-life. However, as reported previously for bovine milk (29, 30) and observed here for the casein and whey fractions of bovine, caprine, and ovine milk samples, heat-treatments affect the activity of the native enzymes. For instance, and as observed here, higher-temperature, short-time heat-treatment (85°C/5 min) reduced to 93–100% the original plasmin activity in both the casein and whey fraction of all ruminants' milk. According to other studies (31, 32), the components of the plasmin system have variable heat sensitivities. During mild heat-treatment (75°C for 15 s), plasminogen inhibitor loses 81% of its original activity, while plasminogen activators are more heat-stable (*D* value of 32 s at 140°C), increasing the conversion of plasminogen to plasmin. Thus, the activation of plasminogen into plasmin can result from the heat sensitivity of the components, which are part of the complex enzyme system. For example, bovine milk has more plasminogen (zymogen) than plasmin activity in the casein fraction; however, when the raw milk was heat-treated at 63°C/30 min, the plasminogen-derived activity decreased (25%), and the plasmin activity increased (100%), suggesting the conversion of the zymogen to active enzyme during this thermal treatment. This appears to explain both the higher number of predicted peptides cleaved by plasmin in the heat-treated bovine milk (103 cleavages) compared to its raw counterpart (45 cleavages) (Figure 4) and the low number of overlapping peptides between heat-treated and raw bovine milk. For instance, the peptide SSRQPQSQNPKLPLSILKEKHL (22 amino acids) identified in raw bovine milk was also identified as 18 (SSRQPQSQNPKLPLSILK) and 10 (LPLSLKEKHL) amino acids at 63°C/30 min.

Similar to the casein fraction of the bovine milk, the plasmin activity of the whey fraction in the ovine milk also increased (45%) after the thermal treatment at 63°C/30 min, but the plasminogen activity was not modified. The reasons for this increase in plasmin activity are unclear.

Mass spectrometry experiments were designed to characterize the endogenous peptides qualitatively. The results were also used to predict the number of peptides cleaved by the main milk proteases. This approach has been used in other studies (25, 33). Care is needed when interpreting the number of cleaved peptides, as it does not account for the total number of peptides in the sample.

The number of non-redundant endogenous peptides identified in this study (72 peptides) using a qualitative peptidomic approach for raw bovine milk is within the range of peptides reported by other researchers: 33 (34) and 159 (35) peptides. However, raw ovine (790 peptides) and caprine (374 peptides) milk showed higher number of peptides than previously reported: 718 (36) and 261 (37) peptides, respectively. The variation in the number of peptides between the studies could be associated with different factors such as the mass spectrometry instrument used, the milk origin, and lactation stage of the milk samples used.

Most of the peptides generated during the heat-treatment of ovine milk at 63°C/30 min were derived from β-casein and were predicted to be mainly hydrolyzed by cathepsin D. This result suggests that although ovine milk had the highest plasmin activity, plasmin was not responsible for most of the hydrolysis that happened during the heat-treatment.

According to the EnzymePredictor tool, the heat-treatment (85°C/5 min) reduced the number of peptide bonds cleaved by plasmin, cathepsin D, and elastase. This reduction can be ascribed to the inactivation of the enzymes during this treatment, which was confirmed when plasmin (active or inactive form) activity was evaluated as a model enzyme. Although the later supports the predicted number of cleaved peptides, further work using quantitative peptides identification is warranted.

Like ovine milk, most of the peptides with potential bioactivity identified in bovine and caprine milk were treated at 63°C/30 min derived from β-casein. However, the predicted hydrolysis was mainly caused by plasmin. Previous studies have reported that most of the peptides in milk are mainly derived from β-casein (35, 38), and this can be ascribed to the structure (primary to quaternary) of this protein, which renders it more susceptible to proteolysis. The hydrolysis of proteins during the thermal treatment releases peptides with different functions than those present in raw milk. For example, ovine milk samples heated at 63°C/30 min have peptides with specific biological activities, such as cytomodulatory and opioid, that were not detected in raw ovine milk samples.

Some of the potential bioactive peptides identified in pasteurized milk, such as with opioid bioactivity, are resistant to gastric conditions (3) and, combined with bioactive peptides released during gastrointestinal digestion, can increase the availability of functional peptides reaching the intestine, where they are then distributed throughout the body by the circulation blood (2). However, future research is needed to determine the bioavailability of the potential bioactive peptides identified in this study.

In conclusion, non-specific and specific protease activities in casein and whey fractions of ruminant milk samples were reduced when high-temperature, long-time (85°C for 5 min) treatment was applied, and this was reflected in the predicted number of peptides broken by the main milk proteases. The use of mild heat-treatments, like 63°C/30 min, preserved the activities of the studied native proteolytic enzymes in ruminants' milk and generated new potential bioactive peptides with different purported functions than those detected in raw ruminant milk samples.

## Data Availability Statement

The original contributions presented in the study are publicly available. This data can be found here: http://proteomecentral.proteomexchange.org/cgi/GetDataset?ID=PXD022702.

## Author Contributions

NR and WM developed the research project and sourced the funding. JL, CM, SL, JM, NR, and WM designed the study. JL performed the experiments. JL supported by SL, CM, and JM analyzed the data. JL supported by CM, JM, NR, EM, SL, and WM wrote and edited the paper. CM did the statistical analysis. EM identified the peptides. All authors contributed to the article and approved the submitted version.

## Conflict of Interest

The authors declare that the research was conducted in the absence of any commercial or financial relationships that could be construed as a potential conflict of interest.
