# Supplementary material for: Heat-Treatments Affect Protease Activities and Peptide Profiles of Ruminants' Milk
Source: Front Nutr. 2021 Mar 10;8:626475. doi: 10.3389/fnut.2021.626475 (PMC7987661; doi:10.3389/fnut.2021.626475)
Supplement: Supplementary file 1 [file Data_Sheet_1.pdf]

Supplementary Table 1 – Common peptide sequences between raw and heated at 63°C for 30 min ruminants' milk (bovine, ovine and caprine).

	Peptide sequence	Mass (Da)	*m/z*	Start[1]	End[2]	Protein name
**Bovine milk** (3 peptides)	TVDMESTEVFTKK	1514	757.87	153	165	αS2-casein
	TVDMESTEVFTK	1386	693.83	153	164	αS2-casein
	AINPSKENLCSTFCK	1652	551.60	42	56	αS2-casein
	TGPIPNSLPQNILPLTQTPVVVPPFLQPEIMGVPK	3731	1244.70	78	112	β-casein
	YPFTGPIPN	986	987.49	75	83	β-casein
	SLSQPKVLPVPQKAVPQRDMPIQAFLL	3000	750.94	179	205	β-casein
	HKEMPFPKYPVEPFTESQSLTLTDVEK	3177	636.32	121	147	β-casein
	EMPFPKYPVEPFTESQSL	2125	709.34	123	140	β-casein
	TPVVVPPFLQPEIMGVPK	1985	662.69	95	112	β-casein
	MPFPKYPVEPFTESQS	1883	942.44	124	139	β-casein
	EPVLGPVRGPFPIL	1533	767.44	208	221	β-casein
	HKEMPFPKYPVEPFTESQSL	2390	598.55	121	140	β-casein
	YQEPVLGPVRGPFPILV	1894	948.04	206	222	β-casein
	FQSEEQQQTEDELQDKIHPFAQA	2825	942.75	48	70	β-casein
	PQNILPLTQTPVVVPPFLQPEIMGVPK	2952	984.89	86	112	β-casein
	PLTQTPVVVPPFLQPEIMGVPKVKE	2854	952.20	91	115	β-casein
	SLSQPKVLPVPQKAVPQRDMPIQA	2626	657.62	179	202	β-casein
	QEPVLGPVRGPFPILV	1755	439.74	207	222	β-casein
	QQTEDELQDK	1275	638.29	54	63	β-casein
	TQTPVVVPPFLQPEIMGVPK	2214	554.55	93	112	β-casein
	SQPKVLPVPQKAVPQRDMPIQAFLL	2816	704.90	181	205	β-casein
	IEKFQSEEQQQTEDELQDKIHPFAQA	3115	779.88	45	70	β-casein
	QSEEQQQTEDELQDK	1914	957.88	49	63	β-casein
	TPVVVPPFLQPEIM	1582	791.93	95	108	β-casein
	FQSEEQQQTEDELQDKIHPFAQAQS	3040	1014.44	48	72	β-casein
	YQEPVLGPVRGPFPIL	1819	607.32	206	221	β-casein
	VPPFLQPEIMGVPKVKE	1907	636.70	99	115	β-casein
	QPPQPLPPTVMFPPQ	1656	828.93	161	175	β-casein
	QPPQPLPP	872	873.48	161	168	β-casein
	EPVLGPVRGPFP	1307	654.36	208	219	β-casein

**Ovine milk** (95 peptides)	KFQSEEQQQTEDELQDK	2109	703.99	47	63	β-casein
	QPKVLPVPQ	1005	503.31	182	190	β-casein
	YQEPVL	747	748.39	206	211	β-casein
	EPVLGPVR	866	433.76	208	215	β-casein
	LPVPQKAVPQRDMPIQ	1816	606.34	186	201	β-casein
	QNILPLTQTPVVVPPFL	1858	930.04	87	103	β-casein
	FTGPIPNSLPQNILPLTQTPVVVPPFLQPEIMGVPK	3878	970.54	77	112	β-casein
	EQEELNVVGETVESLSSSEESITHIN	3018	1510.13	17	42	β-casein
	TDVEKLHLPLPLVQS	1686	562.98	143	157	β-casein
	DMPIQA	689	690.31	197	202	β-casein
	MPFPKYPVEPFTES	1668	834.90	124	137	β-casein
	PLTQTPVVVPPFLQPEIMGVPKVKET	2845	949.20	91	116	β-casein
	VPQRDMPIQAFL	1430	715.88	193	204	β-casein
	VPPFLQPEIMGVPKVKETMVPK	2479	620.85	99	120	β-casein
	EQEELNVV	940	471.23	17	24	β-casein
	QSEEQQQTEDELQDKIHPFAQAQS	2813	938.77	49	72	β-casein
	EELNVVGETVESLSSSEESITHIN	2681	1341.60	19	42	β-casein
	EELNVVGETVESLSSSEESITHINK	2809	937.44	19	43	β-casein
	VQSWMHQPPQPLPPTVMFPPQSV	2643	882.11	155	177	β-casein
	TQTPVVVPPFLQPEIMGVPKVKE	2548	850.47	93	115	β-casein
	LNENKVL	870	1462.68	105	111	β-lactoglobulin
	GTQYTDAPSFSDIPNPIGSENSGKITMP	2923	1387.66	185	212	αS1-casein
	TDAPSFSDIPNPIGSENSGKITMPLW	2773	644.35	189	214	αS1-casein
	NVPQLEIVPKSAEEQLH	1930	609.31	120	136	αS1-casein
	NPIGSENSGKIT	1217	1131.55	199	210	αS1-casein
	YLPLGTQYTDAPSFSDIPNPIGSENSGKITMP	3392	1078.53	181	212	αS1-casein
	SDIPNPIGSENSGKITMPLW	2155	698.70	195	214	αS1-casein
	YNVPQLEIVPKSAEEQLH	2093	576.29	119	136	αS1-casein
	KYNVPQLEIVPKSAEEQLH	2301	903.92	118	136	αS1-casein
	AGSSSSSEEIVPNSAEQK	1806	710.71	77	94	αS1-casein
	KHQGLSPEVLNENLLRF	2129	748.66	22	38	αS1-casein
	SKDIGSESIEDQAMEDAKQM	2243	630.81	56	75	αS1-casein
	KYNVPQLEIVPKSAEEQLHSM	2519	723.35	118	138	αS1-casein

NPIGSENSGKITMP	1445	1065.42	199	212	αS1-casein
AGSSSSSEEIVPNSAEQKY	2129	1195.55	77	95	αS1-casein
DAPSFSDIPNPIGSENSGKITMP	2389	949.93	190	212	αS1-casein
GSSSSSEEIVPNSAEQKY	1898	771.39	78	95	αS1-casein
YNVPQLEIVPKSAEEQLHSM	2311	411.92	119	138	αS1-casein
VLNENLLRFV	1233	493.48	30	39	αS1-casein
LKKYNVPQLEIVPKSAEEQLH	2462	633.85	116	136	αS1-casein
KPWTQPKTNAI	1266	697.80	207	217	αS2-casein
DMESTEVFTKK	1394	641.75	156	166	αS2-casein
DMESTEVFTK	1281	643.11	156	165	αS2-casein
VNREQLSTSEENSKKTIDMESTEVFTK	3210	1154.39	139	165	αS2-casein
DMESTEVFT	1153	943.41	156	164	αS2-casein
SIRSSSEESAEVAPEEV	1885	987.40	69	85	αS2-casein
CTTSCEEVVRNANEEEY	1973	655.28	52	68	αS2-casein
CTTSCEEVVRNA	1309	562.74	52	63	αS2-casein
CTTSCEEVVR	1123	703.68	52	61	αS2-casein
SSSEEPINISQEIYKQEK	2108	639.30	23	40	αS2-casein
EHVSSSEEPINISQEIYKQEK	2553	676.06	20	40	αS2-casein
MEHVSSSEEPINISQEIYKQEK	2700	574.89	19	40	αS2-casein
HKMEHVSSSEEPINISQEIYKQEK	2869	648.49	17	40	αS2-casein
KHKMEHVSSSEEPINISQEIYKQEK	3237	809.70	16	40	αS2-casein
EHVSSSEEPINISQEIYKQE	2426	686.33	20	39	αS2-casein
HKMEHVSSSEEPINISQEIYKQE	2741	723.67	17	39	αS2-casein
EHVSSSEEPINISQEIYK	2168	1639.21	20	37	αS2-casein
VVNAVDNPEASSESIASAPETNTAQVTSTEV	3276	1403.15	162	192	κ-casein
AVDNPEASSESIASAPETNTAQVTSTEV	2804	1447.60	165	192	κ-casein
VDNPEASSESIASAPETNTAQVTSTEV	2893	1540.14	166	192	κ-casein
NAVDNPEASSESIASAPETNTAQVTSTEV	3078	1243.54	164	192	κ-casein
PEASSESIASAPETNTAQVTSTEV	2485	407.46	169	192	κ-casein
DSVSSEVN	915	572.28	271	278	Osteopontin
DPNHFRPAGLPNKY	1626	526.05	134	147	Serum amyloid A protein
GPGGVW	571	436.25	65	70	Serum amyloid A protein
GMTRDQVREDTKADQFANEWGR	2625	916.33	92	113	Serum amyloid A protein

	RPAGLPNKY	1016	508.78	139	147	Serum amyloid A protein
	GNYDAAQRGPGGVW	1445	723.33	57	70	Serum amyloid A protein
**Caprine milk** (22 peptides)	INHQGLSPEVPNENLL	1773	591.98	20	35	αs1-casein
	KYNVPQLEIVPKSAEEQLHSM	2519	630.81	117	137	αs1-casein
	QGLSPEVLNENLLR	1581	791.44	24	37	αs1-casein
	IDMESTEVFTK	1379	690.30	155	165	αS2-casein
	KTIDMESTEVFTK	1608	536.92	153	165	αS2-casein
	RNAGPFTPTVNREQLSTSEENS	2513	838.71	130	151	αS2-casein
	TIDMESTEVFTK	1496	748.82	154	165	αS2-casein
	QEPVLGPVR	977	977.54	207	215	β-casein
	QEPVLGPVRGPFP	1375	688.37	207	219	β-casein
	QEPVLGPVRGPFPI	1488	744.92	207	220	β-casein
	QEPVLGPVRGPFPIL	1601	801.46	207	221	β-casein
	REQEELNVVG	1172	586.80	16	25	β-casein
	REQEELNVVGETVESLSSSEESITHIN	3094	1032.47	16	42	β-casein
	REQEELNVVGETVESLSSSEESITHINK	3302	826.62	16	43	β-casein
	REQEELNVVGETVESLSSSEESITHINKKIE	3593	719.55	16	46	β-casein
	YQEPVLGPVRGPFPILV	1918	480.51	206	222	β-casein
	QNQNPKLPLSILKEKQL	1990	498.55	78	94	Glycosylation-dependent cell adhesion molecule 1
	ETNTAQVTSTEV	1359	680.29	181	192	κ-casein
	IVNTVDNPEASSES	1541	771.32	162	175	κ-casein
	DQFANEWGR	1121	561.75	106	114	Serum amyloid A protein
	KDPNHFGPADLPDKY	1713	571.95	114	128	Serum amyloid A protein
	RGPGGAWAAKVISNA	1454	485.60	65	79	Serum amyloid A protein

[1]**Start:** Starting position of peptide in the complete protein sequence.

[2]**End:** Ending position peptide in the complete protein sequence.

Supplementary Table 2 – Common peptide sequences between raw and heated at 85°C for 5 min ruminants' milk (bovine, ovine and caprine).

	Peptide sequence	Mass (Da)	*m/z*	Start[1]	End[2]	Protein name
**Bovine milk** (5 peptides)	QEPVLGPVR	977	977.54	209	217	β-casein
	TVDMESTEVFTK	1402	701.82	153	164	αS2-casein
	PEVIESPPEINTVQVTSTAV	2109	1055.55	171	190	κ-casein
	QPQSQNPKLPLSIL	1545	773.44	75	88	Glycosylation-dependent cell adhesion molecule 1
	SSRQPQSQNPKLPLSILKEK	2259	452.86	72	91	Glycosylation-dependent cell adhesion molecule 1
**Ovine milk** (41 peptides)	QNILPLTQTPVVVPPFLQPEIMGVPK	2838	1419.79	87	112	β-casein
	QPKVLPVPQK	1116	558.84	182	191	β-casein
	QPPQPLPPTVMFPPQSV	1842	921.98	161	177	β-casein
	EQEELNVVGETVESLSSSEESITHIN	2858	1430.17	17	42	β-casein
	TQTPVVVPPFLQPEIMGVPK	2192	731.74	93	112	β-casein
	IEKFQSEEQQQTEDELQDK	2351	784.70	45	63	β-casein
	LTQTPVVVPPFLQPEIMGVPK	2305	769.43	92	112	β-casein
	KIEKFQSEEQQQTEDELQDKIHPF	2973	595.70	44	67	β-casein
	TGPIPNSLPQNILPLTQTPVVVPPFLQPEIMGVPK	3747	1250.03	78	112	β-casein
	MPFPKYPVEP	1204	602.81	124	133	β-casein
	PLTQTPVVVPPFLQPEIMGVPKV	2515	1258.71	91	113	β-casein
	PLTQTPVVVPPFLQPEI	1874	938.03	91	107	β-casein
	QPPQPLPPTVMFPPQS	1776	888.95	161	176	β-casein
	QSEEQQQTEDELQDK	1818	909.90	49	63	β-casein
	QNILPLTQTPVVVPPFLQPEIMGVPKVKETMVPK	3799	950.78	87	120	β-casein
	SWMHQPPQPLPPTVMFPPQSV	2400	801.07	157	177	β-casein
	PQNILPLTQTPVVVPPFLQPEI	2440	1221.20	86	107	β-casein
	NPIGSENSGKITMP	1444	722.86	199	212	αS1-casein
	YLPLGTQYTDAPSFSDIPNPIGSENSGKITMP	3426	1142.88	181	212	αS1-casein
	TDAPSFSDIPNPIGSENSGKITMP	2474	1238.08	189	212	αS1-casein
	LPLGTQYTDAPSFSDIPNPIGSENSGKITMP	3263	1088.53	182	212	αS1-casein
	IPNPIGSENSGKITMP	1654	827.93	197	212	αS1-casein
	GSSSSSEEIVPNSAEQKY	1978	989.92	78	95	αS1-casein
	AGSSSSSEEIVPNSAEQK	1886	943.91	77	94	αS1-casein
	VNREQLSTSEENSKKTIDMESTEVFTK	3210	643.11	139	165	αS2-casein

	DMESTEVFT	1073	1074.43	156	164	αS2-casein
	SIRSSSEESAEVAPEEV	1805	903.42	69	85	αS2-casein
	CTTSCEEVVR	1123	562.74	52	61	αS2-casein
	MEHVSSSEEPINISQEIYKQEK	2684	672.06	19	40	αS2-casein
	HKMEHVSSSEEPINISQEIYKQEK	2885	578.09	17	40	αS2-casein
	KHKMEHVSSSEEPINISQEIYKQEK	3157	632.49	16	40	αS2-casein
	EHVSSSEEPINISQEIYK	2168	723.67	20	37	αS2-casein
	EHVSSSEEPINISQE	1764	882.87	20	34	αS2-casein
	EHVSSSEEPINI	1340	670.82	20	31	αS2-casein
	VVNAVDNPEASSESIASAPETNTAQVTSTEV	3196	1599.22	162	192	κ-casein
	PEASSESIASAPETNTAQVTSTEV	2565	1283.53	169	192	κ-casein
	NTAQVTSTEV	1128	1129.47	183	192	κ-casein
	AVDNPEASSESIASAPETNTAQVTSTEV	2884	1443.13	165	192	κ-casein
	VDNPEASSESIASAPETNTAQVTSTEV	2893	1447.58	166	192	κ-casein
	SNVESPDATEEDF	1519	1519.55	170	182	Osteopontin
	NVESPDATEEDF	1352	1352.55	171	182	Osteopontin
**Caprine milk** (10 peptides)	YNVPQLEIVPK	1313	1313.72	118	128	αS1-casein
	KTIDMESTEVFTK	1624	812.86	153	165	αS2-casein
	KIEKFQSEEQQQTEDELQDK	2559	1280.56	44	63	β-casein
	KKIEKFQSEEQQQTEDELQD	2560	1281.07	43	62	β-casein
	QEELNVVG	886	887.45	18	25	β-casein
	REQEELNVVGETVESLSSSEESITHINK	3222	1075.17	16	43	β-casein
	QDENYEEAIVHLK	1570	785.87	128	140	Butyrophilin subfamily 1 member A1
	QNQNPKLPLSILKE	1621	541.31	78	91	Glycosylation-dependent cell adhesion molecule 1
	KDSKADQFANEWG	1533	767.32	101	113	Serum amyloid A protein
	REAGQGAKDMW	1264	632.79	26	36	Serum amyloid A protein

[1]**Start:** Starting position of peptide in the complete protein sequence.

[2]**End:** Ending position peptide in the complete protein sequence.

Supplementary Table 3 - Peptides found in each treatment (raw, 63°C/30 min and 85°C/5 min) and species (bovine, ovine and caprine) combination that have an exact match with the bioactive peptides database (MBPDB)

	Treatment	Peptide	Protein description	Function	Reference
**Bovine milk**	Raw (3 peptides)	LPQNIPPLT	β-casein	DPP-IV inhibitory	Nongonierma and FitzGerald (2016)
		LPLP	β-casein	ACE-inhibitory	Kohmura et al. (1990)
		TPEVDDEALEK	β-lactoglobulin	DPP-IV inhibitory	Silveira et al. (2013)
	63°C/30 min (5 peptides)	FPEVFGK	αS1-casein	ACE-inhibitory	Maruyama et al. (1987)
		RELEELNVPGEIVESLSSSEESITR	β-casein	Immunomodulatory	Hata et al. (1998)
		VLPVPQKAVPYPQR	β-casein	Antimicrobial	Sedaghati et al. (2016)
		EPVLGPVRGPFP	β-casein	ACE-inhibitory	Hayes et al. (2007)
		LIVTQTMK	β-lactoglobulin	Cytotoxic	Jacquot (2010)
	85°C/5 min (3 peptides)	QMEAESISSSEEIVPNSVEQK	αS1-casein	Immunomodulatory	Hata et al. (1998)
		NMAINPSKENLCSTFCK	αS2-casein	ACE-inhibitory	Tu et al. (2018)
		RELEELNVPGEIVESLSSSEESITR	β-casein	Immunomodulatory	Hata et al. (1998)
	Raw (26 peptides)	DAYPSGAW	αS1-casein	ACE-inhibitory	Pihlanto-Leppälä et al. (1998)
		RYL	αS1-casein	Antioxidant	Contreras et al. (2013)
		VLNENLLR	αS1-casein	Antimicrobial	Hayes et al. (2006)
		YLEQLLR	αS1-casein	Antimicrobial	Liu et al. (2015)
		YLGYLEQ	αS1-casein	Anxiolytic	Cakir-Kiefer et al. (2011)
		VPSERYL	αS1-casein	ACE-inhibitory	Ruiz et al. (2004)
		NPWDQ	αS2-casein	Immunomodulatory	Isobe et al. (2008)
		VRYL	αS2-casein	ACE-inhibitory	Ruiz et al. (2004)
		YQKFPQY	αS2-casein	ACE-inhibitory	Silva et al. (2006)
		HLPLP	β-casein	ACE-inhibitory	Kohmura et al. (1990)
		LPLP	β-casein	ACE-inhibitory	Kohmura et al. (1990)
		EPVLGPVRGPFP	β-casein	ACE-inhibitory	Hayes et al. (2007)
		GPFPI	β-casein	Cathepsin B Inhibitory	Lee and Lee (2000)
		LPVP	β-casein	DPP-IV inhibitory	Nongonierma et al. (2018)
		LPVPQ	β-casein	DPP-IV inhibitory	Nongonierma and FitzGerald (2016)
		MPFPKYPVEP	β-casein	ACE-inhibitory	Hayes et al. (2007)
		YQEPVLGP	β-casein	Antioxidant	Silva et al. (2006)

		YQEPVLGPVRGPFPI	β-casein	Antimicrobial	Almaas et al. (2011)
		TPVVVPPFLQP	β-casein	ACE-inhibitory	Abubakar et al. (1998)
		VLPVPQK	β-casein	Antioxidant	Shanmugam et al. (2015)
		YPVEPFTE	β-casein	Bradykinin-Potentiating	Perpetuo et al. (2003)
		YQEPVL	β-casein	ACE-inhibitory	Pihlanto-Leppälä et al. (1998)
		HPHPHLSF	κ-casein	ACE-inhibitory	Gómez-Ruiz et al. (2007)
		IPIQY	κ-casein	ACE-inhibitory	Nongonierma and FitzGerald (2014)
**Ovine milk**		YIPIQY	κ-casein	DPP-IV inhibitory	Gómez-Ruiz et al. (2007)
		LRP	Lactotransferrin	ACE-inhibitory	García-Tejedor et al. (2014)
	63°C/30 min (15 peptides)	FPQYLQY	αS2-casein	ACE-inhibitory	Tauzin et al. (2002)
		FPQY	αS2-casein	ACE-inhibitory	Contreras et al. (2013)
		EPVLGPVRGPFP	β-casein	ACE-inhibitory	Hayes et al. (2007)
		YQEPVL	β-casein	ACE-inhibitory	Pihlanto-Leppälä et al. (1998)
		QSLVYP	β-casein	ACE-inhibitory	Kohmura et al. (1990)
		EPVLGPVRGP	β-casein	Cytomodulatory	Zhao et al. (2014)
		LPQNILP	β-casein	ACE-inhibitory	Kohmura et al. (1990)
		LPLPL	β-casein	DPP-IV inhibitory	Nongonierma and FitzGerald (2014)
		YPVEPF	β-casein	DPP-IV inhibitory	Nongonierma and FitzGerald (2016)
		QEPVL	β-casein	immunomodulatory	Jiehui et al. (2014)
		YQEPVLGPVR	β-casein	Immunomodulatory	Kayser and Meisel (1996)
		YPVEPF	β-casein	Opioid	Plaisancié et al. (2015)
		IYP	β-casein	ACE-inhibitory	Kohmura et al. (1990)
		IIVTQTMK	β-lactoglobulin	Antimicrobial	Almaas et al. (2011)
		YPSYGLN	κ-casein	Opioid	Meisel et al. (1989)
	85°C/5 min (7 peptides)	GPFPILV	β-casein	DPP-IV inhibitory	Zhang et al. (2016)
		LVYP	β-casein	ACE-inhibitory	Kohmura et al. (1990)
		LVYPFTGPIPN	β-casein	ACE-inhibitory	Quirós et al. (2005)
		MPFPKYPVEP	β-casein	ACE-inhibitory	Hayes et al. (2007)
		QSLVYP	β-casein	ACE-inhibitory	Kohmura et al. (1990)
		REQEELNV	β-casein	Antimicrobial	Kohmura et al. (1990)
		TQTPVVVPPFLQPE	β-casein	Antioxidant	Sabeena Farvin et al. (2010)
	Raw	DAYPSGAW	αS1-casein	ACE-inhibitory	Pihlanto-Leppälä et al. (1998)

	(12 peptides)	VLNENLLR	αS1-casein	Antimicrobial	Hayes et al. (2006)
		EPVLGPVRGPFP	β-casein	ACE-inhibitory	Hayes et al. (2007)
		GPFPILV	β-casein	DPP-IV inhibitory	Zhang et al. (2016)
		KIHPFAQAQ	β-casein	ACE-inhibitory	Quirós et al. (2005)
		REQEELNV	β-casein	Antimicrobial	Almaas et al. (2011)
		YQEPVL	β-casein	ACE-inhibitory	Pihlanto-Leppälä et al. (1998)
**Caprine milk**		YQEPVLGP	β-casein	Antioxidant	Silva et al. (2006)
		YQEPVLGPVR	β-casein	Immunomodulatory	Lu et al. (2016)
		YQEPVLGPVRGPFPI	β-casein	Antimicrobial	Almaas et al. (2011)
		YIPIQY	κ-casein	ACE-inhibitory	Gómez-Ruiz et al. (2007)
		YVL	κ-casein	Antioxidant	López-Expósito et al. (2007)
	63°C/30 min (4 peptides)	QEPVL	β-casein	Immunomodulatory	Jiehui et al. (2014)
		LVYP	β-casein	ACE-inhibitory	Kohmura et al. (1990)
		VLPVPQK	β-casein	Antioxidant	Shanmugam et al. (2015)
		TQTPVVVPPFLQPE	β-casein	Antioxidant	Sabeena Farvin et al. (2010)
	85°C/5 min (1 peptide)	LVYPFTGPIPN	β-casein	ACE-inhibitory	Quirós et al. (2005)

References

Abubakar, A., Saito, T., Kitazawa, H., Kawai, Y., and Itoh, T. (1998). Structural Analysis of New Antihypertensive Peptides Derived from Cheese Whey Protein by Proteinase K Digestion. *Journal of Dairy Science* 81(12)**,** 3131-3138. doi: https://doi.org/10.3168/jds.S0022-0302(98)75878-3.

Almaas, H., Eriksen, E., Sekse, C., Comi, I., Flengsrud, R., Holm, H., et al. (2011). Antibacterial peptides derived from caprine whey proteins, by digestion with human gastrointestinal juice. *British Journal of Nutrition* 106(6)**,** 896-905. doi: 10.1017/S0007114511001085.

Cakir-Kiefer, C., Le Roux, Y., Balandras, F., Trabalon, M., Dary, A., Laurent, F., et al. (2011). In Vitro Digestibility of α-Casozepine, a Benzodiazepine-like Peptide from Bovine Casein, and Biological Activity of Its Main Proteolytic Fragment. *Journal of Agricultural and Food Chemistry* 59(9)**,** 4464-4472. doi: 10.1021/jf104089c.

Contreras, M.d.M., Sanchez, D., Sevilla, M.Á., Recio, I., and Amigo, L. (2013). Resistance of casein-derived bioactive peptides to simulated gastrointestinal digestion. *International Dairy Journal* 32(2)**,** 71-78. doi: https://doi.org/10.1016/j.idairyj.2013.05.008.

García-Tejedor, A., Sánchez-Rivera, L., Castelló-Ruiz, M., Recio, I., Salom, J.B., and Manzanares, P. (2014). Novel antihypertensive lactoferrin-derived peptides produced by Kluyveromyces marxianus: gastrointestinal stability profile and in vivo angiotensin I-converting enzyme (ACE) inhibition. *J Agric Food Chem* 62(7)**,** 1609-1616. doi: 10.1021/jf4053868.

Gómez-Ruiz, J.Á., Ramos, M., and Recio, I. (2007). Identification of novel angiotensin-converting enzyme-inhibitory peptides from ovine milk proteins by CE-MS and chromatographic techniques. *ELECTROPHORESIS* 28(22)**,** 4202-4211. doi: 10.1002/elps.200700324.

Hata, I., Higashiyama, S., and Otani, H. (1998). Identification of a phosphopeptide in bovine alpha s1-casein digest as a factor influencing proliferation and immunoglobulin production in lymphocyte cultures. *The Journal of dairy research* 65(4)**,** 569-578. doi: 10.1017/s0022029998003136.

Hayes, M., Ross, R.P., Fitzgerald, G.F., Hill, C., and Stanton, C. (2006). Casein-derived antimicrobial peptides generated by Lactobacillus acidophilus DPC6026. *Appl Environ Microbiol* 72(3)**,** 2260-2264. doi: 10.1128/aem.72.3.2260-2264.2006.

Hayes, M., Stanton, C., Slattery, H., O'Sullivan, O., Hill, C., Fitzgerald, G.F., et al. (2007). Casein Fermentate of Lactobacillus animalis DPC6134 Contains a Range of Novel Propeptide Angiotensin-Converting Enzyme Inhibitors. *Applied and Environmental Microbiology* 73(14)**,** 4658-4667. doi: 10.1128/aem.00096-07.

Isobe, N., Suzuki, M., Oda, M., and Tanabe, S. (2008). Enzyme-Modified Cheese Exerts Inhibitory Effects on Allergen Permeation in Rats Suffering from Indomethacin-Induced Intestinal Inflammation. *Bioscience, Biotechnology, and Biochemistry* 72(7)**,** 1740-1745. doi: 10.1271/bbb.80042.

Jacquot, A. (2010). Proliferative effects of synthetic peptides from β-lactoglobulin and α-lactalbumin on murine splenocytes. *International dairy journal* v. 20(no. 8)**,** pp. 514-521-2010 v.2020 no.2018. doi: 10.1016/j.idairyj.2010.02.013.

Jiehui, Z., Liuliu, M., Haihong, X., Yang, G., Yingkai, J., Lun, Z., et al. (2014). Immunomodulating effects of casein-derived peptides QEPVL and QEPV on lymphocytes in vitro and in vivo. *Food & Function* 5(9)**,** 2061-2069. doi: 10.1039/C3FO60657K.

Kayser, H., and Meisel, H. (1996). Stimulation of human peripheral blood lymphocytes by bioactive peptides derived from bovine milk proteins. *FEBS Letters* 383(1-2)**,** 18-20. doi: 10.1016/0014-5793(96)00207-4.

Kohmura, M., Nio, N., and Ariyoshi, Y. (1990). Inhibition of Angiotensin-Converting Enzyme by Synthetic Peptide Fragments of Various β-Caseins. *Agricultural and Biological Chemistry* 54(4)**,** 1101-1102. doi: 10.1271/bbb1961.54.1101.

Lee, H.S., and Lee, K.J. (2000). Cathepsin B inhibitory peptides derived from β-casein. *Peptides* 21(6)**,** 807-809. doi: https://doi.org/10.1016/S0196-9781(00)00212-6.

Liu, Y., Eichler, J., and Pischetsrieder, M. (2015). Virtual screening of a milk peptide database for the identification of food-derived antimicrobial peptides. *Molecular Nutrition & Food Research* 59(11)**,** 2243-2254. doi: 10.1002/mnfr.201500182.

López-Expósito, I., Quirós, A., Amigo, L., and Recio, I. (2007). Casein hydrolysates as a source of antimicrobial, antioxidant and antihypertensive peptides. *Lait* 87(4-5)**,** 241-249.

Lu, Y., Govindasamy-Lucey, S., and Lucey, J.A. (2016). Angiotensin-I-converting enzyme-inhibitory peptides in commercial Wisconsin Cheddar cheeses of different ages. *Journal of Dairy Science* 99(1)**,** 41-52. doi: https://doi.org/10.3168/jds.2015-9569.

Maruyama, S., Mitachi, H., Tanaka, H., Tomizuka, N., and Suzuki, H. (1987). Studies on the Active Site and Antihypertensive Activity of Angiotensin I-Converting Enzyme Inhibitors Derived from Casein. *Agricultural and Biological Chemistry* 51(6)**,** 1581-1586. doi: 10.1080/00021369.1987.10868244.

Meisel, H., Frister, H., and Schlimme, E. (1989). Biologically active peptides in milk proteins. *Zeitschrift für Ernährungswissenschaft* 28(4)**,** 267-278. doi: 10.1007/BF02019390.

Nongonierma, A.B., and FitzGerald, R.J. (2014). Susceptibility of milk protein-derived peptides to dipeptidyl peptidase IV (DPP-IV) hydrolysis. *Food Chemistry* 145**,** 845-852. doi: https://doi.org/10.1016/j.foodchem.2013.08.097.

Nongonierma, A.B., and FitzGerald, R.J. (2016). Structure activity relationship modelling of milk protein-derived peptides with dipeptidyl peptidase IV (DPP-IV) inhibitory activity. *Peptides* 79**,** 1-7. doi: https://doi.org/10.1016/j.peptides.2016.03.005.

Nongonierma, A.B., Paolella, S., Mudgil, P., Maqsood, S., and FitzGerald, R.J. (2018). Identification of novel dipeptidyl peptidase IV (DPP-IV) inhibitory peptides in camel milk protein hydrolysates. *Food Chem* 244**,** 340-348. doi: 10.1016/j.foodchem.2017.10.033.

Perpetuo, E.A., Juliano, L., and Lebrun, I. (2003). Biochemical and Pharmacological Aspects of Two Bradykinin-Potentiating Peptides Obtained from Tryptic Hydrolysis of Casein. *Journal of Protein Chemistry* 22(7)**,** 601-606. doi: 10.1023/B:JOPC.0000008724.98339.ff.

Pihlanto-Leppälä, A., Rokka, T., and Korhonen, H. (1998). Angiotensin I Converting Enzyme Inhibitory Peptides Derived from Bovine Milk Proteins. *International Dairy Journal* 8(4)**,** 325-331. doi: https://doi.org/10.1016/S0958-6946(98)00048-X.

Plaisancié, P., Boutrou, R., Estienne, M., Henry, G., Jardin, J., Paquet, A., et al. (2015). β-Casein(94-123)-derived peptides differently modulate production of mucins in intestinal goblet cells. *Journal of Dairy Research* 82(1)**,** 36-46. doi: 10.1017/S0022029914000533.

Quirós, A., Hernández-Ledesma, B., Ramos, M., Amigo, L., and Recio, I. (2005). Angiotensin-Converting Enzyme Inhibitory Activity of Peptides Derived from Caprine Kefir. *Journal of Dairy Science* 88(10)**,** 3480-3487. doi: https://doi.org/10.3168/jds.S0022-0302(05)73032-0.

Ruiz, J.Á.G., Ramos, M., and Recio, I. (2004). Angiotensin converting enzyme-inhibitory activity of peptides isolated from Manchego cheese. Stability under simulated gastrointestinal digestion. *International Dairy Journal* 14(12)**,** 1075-1080. doi: https://doi.org/10.1016/j.idairyj.2004.04.007.

Sabeena Farvin, K.H., Baron, C.P., Nielsen, N.S., Otte, J., and Jacobsen, C. (2010). Antioxidant activity of yoghurt peptides: Part 2 – Characterisation of peptide fractions. *Food Chemistry* 123(4)**,** 1090-1097. doi: https://doi.org/10.1016/j.foodchem.2010.05.029.

Sedaghati, M., Kobarfard, F., Ezzatpanah, H., Boojar, M.M.A., and Ebrahimi, M.T. (2016). Isolation and identification of some antibacterial peptides in the plasmin-digest of β-casein. *Lebensmittel-Wissenschaft + [i.e. und] Technologie. Food science + technology. Science + technologie alimentaire* 68**,** 217-225. doi: 10.1016/j.lwt.2015.12.019.

Shanmugam, V.P., Kapila, R., Kapila, S., and Sonfack, T.K. (2015). Antioxidative peptide derived from enzymatic digestion of buffalo casein. *International dairy journal* 42**,** 1-5. doi: 10.1016/j.idairyj.2014.11.001.

Silva, S.V., Pihlanto, A., and Malcata, F.X. (2006). Bioactive Peptides in Ovine and Caprine Cheeselike Systems Prepared with Proteases from Cynara cardunculus. *Journal of Dairy Science* 89(9)**,** 3336-3344. doi: https://doi.org/10.3168/jds.S0022-0302(06)72370-0.

Silveira, S.T., Martínez-Maqueda, D., Recio, I., and Hernández-Ledesma, B. (2013). Dipeptidyl peptidase-IV inhibitory peptides generated by tryptic hydrolysis of a whey protein concentrate rich in β-lactoglobulin. *Food Chem* 141(2)**,** 1072-1077. doi: 10.1016/j.foodchem.2013.03.056.

Tauzin, J., Miclo, L., and Gaillard, J.-L. (2002). Angiotensin-I-converting enzyme inhibitory peptides from tryptic hydrolysate of bovine αS2-casein. *FEBS Letters* 531(2)**,** 369-374. doi: 10.1016/s0014-5793(02)03576-7.

Tu, M., Wang, C., Chen, C., Zhang, R., Liu, H., Lu, W., et al. (2018). Identification of a novel ACE-inhibitory peptide from casein and evaluation of the inhibitory mechanisms. *Food Chem* 256**,** 98-104. doi: 10.1016/j.foodchem.2018.02.107.

Zhang, Y., Fanglei, Z., Huiqin, M., Ran, C., Shangwu, C., and Yanchun, Z. (2016). Comparison of dipeptidyl peptidase IV-inhibitory activity of peptides from bovine and caprine milk casein by in silico and in vitro analyses. *International dairy journal* 53**,** 37-44. doi: 10.1016/j.idairyj.2015.10.001.

Zhao, H., Zhou, F., Wang, L., Fengling, B., Dziugan, P., Walczak, P., et al. (2014). Characterization of a bioactive peptide with cytomodulatory effect released from casein. *European Food Research and Technology* 238(2)**,** 315-322. doi: 10.1007/s00217-013-2106-7.
